# Phylogeny and Biogeography of Cyanobacteria and Their Produced Toxins

**DOI:** 10.3390/md11114350

**Published:** 2013-11-01

**Authors:** Cristiana Moreira, Vitor Vasconcelos, Agostinho Antunes

**Affiliations:** 1 CIIMAR/CIMAR, Interdisciplinary Centre of Marine and Environmental Research, University of Porto, Rua dos Bragas, 289, Porto 4050-123, Portugal; E-Mails: cmoreira@ciimar.up.pt (C.M.); vmvascon@fc.up.pt (V.V.); 2 Biology Department, Faculty of Sciences, University of Porto, Rua do Campo Alegre, Porto 4169-007, Portugal

**Keywords:** cyanobacteria, cyanotoxins, phylogeny and biogeography

## Abstract

Phylogeny is an evolutionary reconstruction of the past relationships of DNA or protein sequences and it can further be used as a tool to assess population structuring, genetic diversity and biogeographic patterns. In the microbial world, the concept that everything is everywhere is widely accepted. However, it is much debated whether microbes are easily dispersed globally or whether they, like many macro-organisms, have historical biogeographies. Biogeography can be defined as the science that documents the spatial and temporal distribution of a given taxa in the environment at local, regional and continental scales. Speciation, extinction and dispersal are proposed to explain the generation of biogeographic patterns. Cyanobacteria are a diverse group of microorganisms that inhabit a wide range of ecological niches and are well known for their toxic secondary metabolite production. Knowledge of the evolution and dispersal of these microorganisms is still limited, and further research to understand such topics is imperative. Here, we provide a compilation of the most relevant information regarding these issues to better understand the present state of the art as a platform for future studies, and we highlight examples of both phylogenetic and biogeographic studies in non-symbiotic cyanobacteria and cyanotoxins.

## 1. Introduction

### 1.1. Cyanobacteria

Cyanobacteria are a diverse group of microorganisms that have inhabited our planet for nearly 2.5 billion years [1]. Distributed in all types of ecological niches from the cold of Antarctica to the hot springs of Japan, New Zealand and Italy [2,3], they are characterized as being oxygenic photosynthetic prokaryotic microorganisms that possess chlorophyll *a* [4,5]. Cyanobacteria can pose serious risks to aquatic waterways and their users through the production of dense blooms in eutrophic systems leading to a decrease in water quality by the release of off-flavors, toxins, water discoloration and accumulation of surface scums [6]. With more than 1000 species and 150 genera [7] described so far, cyanobacteria are important fixers of atmospheric nitrogen in aquatic ecosystems. They produce oxygen as a by-product of this process [8] and have recently been proposed as a source of hydrogen for fuel production [9]. Cyanobacteria were initially classified as algae and consequently, grouped with plants according to the Botanical Code criteria [10]. Under this type of classification, morphological and physiological characteristics were used [11]. Later, cyanobacteria were reclassified by applying bacteriological criteria that uses morphological and reproductive characteristics; however, this is not supported by recent studies that use molecular data and phylogenetic analyses to revise cyanobacteria taxonomy [12,13,14]. Under the bacteriological code, cyanobacteria are classified into five subsections [4,8]. Subsection I (Order Chroococcales) is comprised of unicellular cyanobacteria with reproduction by binary fission or budding [4,8]. Subsection II (Order Pleurocapsales) is composed of unicellular cyanobacteria that reproduce by multiple fission releasing baeocytes [4,8]. Subsection III (Order Oscillatoriales) is composed of filamentous cyanobacteria, consisting solely of vegetative cells that can undergo binary fission only in a single plane and the formation of hormogonia [4,8]. In subsections IV (Order Nostocales) and V (Order Stigonematales), the cyanobacteria are also filamentous but differ from subsection III by the presence of differentiation cells known as heterocysts and akinetes and the formation of hormogonia [8]. The main difference between subsection IV and V is the form of vegetative reproduction. While in subsection IV the cyanobacteria species divide exclusively by binary fission in one plane (false branching), in subsection V, they divide in more than one plane (true branching) [4,8].

### 1.2. Cyanotoxins

Cyanotoxins are a structurally diverse group of compounds that are produced by cyanobacteria. They are synthesized via a nonribosomal pathway and are a product of the secondary metabolism of cyanobacteria, and thus are not used in their primary metabolism [15,16]. Cyanotoxins occur in several genera of cyanobacteria and have the potential to impact aquatic ecosystems due to their toxic nature to both humans and aquatic animals as well as to wild and livestock animals [15]. Cyanotoxins, due to their toxic properties and mode of action, are classified as hepatotoxins (microcystins, nodularins and cylindrospermopsin), neurotoxins (anatoxin-a, saxitoxins) and dermal toxins (lyngbyatoxin, aplysiatoxin) [17]. Their production is not strain specific and can be found in a diverse range of species. Of all the cyanotoxins that have been described, the most widely studied are the hepatotoxins, microcystins and cylindrospermopsin. Microcystins have a worldwide distribution (found in all of the main continents) and are produced by a variety of cyanobacteria genera (e.g., *Microcystis*, *Nostoc*, *Anabaena*, *Planktothrix*). The relevance of studying them is reinforced by the well-known case of human mortality in a dialysis center in Brazil where 60 patients died of acute liver failure due to water contaminated with this toxin [18]. Cylindrospermopsin has been reported in four of the five continents (Africa is the exception) and in nine different species so far (*Cylindrospermopsis raciborskii*, *Umezakia natans*, *Chrysosporum ovalisporum*, *Chrysosporum bergii*, *Raphidiopsis curvata*, *R. mediterranea*, *Aphanizomenon flos-aquae*, *Anabaena lapponica*, *Lyngbya wollei*), clearly indicating that its production is not species-specific [19,20]. Cylindrospermopsin is known for the outbreak of hepatoenteritis that occurred in Palm Island, Australia in 1979 [21] where 148 people were affected and required hospitalization [22].

Biosynthesis of these compounds is performed by a family of multi-enzymatic complexes called nonribosomal peptide synthetases and polyketide synthases organized into repeated functional units known as modules [23,24]. Its transcription and translation is independent of the messenger RNA and are organized in the genome into gene clusters. These encode enzymes for the biosynthesis, regulation, and export of the toxins. The first biosynthetic pathway to be sequenced was that of microcystins [25], followed by the nodularin [26], cylindrospermopsin and saxitoxin gene clusters [27,28], and more recently, the anatoxin-a gene cluster [29].

## 2. Molecular Studies in Cyanobacteria and Cyanotoxins

Molecular studies in cyanobacteria and their produced cyanotoxins started in the early 1990s. They were based on the detection, identification, quantification, profiling, and assessing of genetic diversity and phylogenetics of a given species or taxa. Initially, detection, identification and enumeration of cyanobacteria were conducted with microscopic techniques, which are based on morphological criteria that ultimately require the presence of an experienced observer. Presence of cyanotoxins is nowadays evaluated by chemical methods such as High-Performance Liquid Chromatography (HPLC) and by immunological assays such as the Enzyme-Linked Immunosorbent Assay (ELISA), tests that not only detect the cyanotoxin but also quantify its concentration in a given sample. Newer and faster tools are necessary and in this sense, molecular methodologies have emerged. They have the advantages, in comparison with traditional methodologies, of being highly specific, sensitive and more rapid. Nowadays, molecular tools applied to cyanobacteria and cyanotoxin investigations are widely used and can be divided into Polymerase Chain Reaction (PCR) and non-PCR based methods. So far, several molecular methods have been developed, which have been applied to cyanobacteria and cyanotoxin research, which include PCR, multiplex-PCR, Restriction Fragment Length Polimorphism (RFLP), Amplified Fragment Length Polymorphism (AFLP), Multilocus Sequence Typing (MLST), Random Amplified Polymorphic DNA (RAPD), Denaturing Gradient Gel Electrophoresis (DGGE), Fluorescence *in situ* Hybridization (FISH), Real-time PCR (qPCR) and DNA microarrays. Their main applications include field investigations regarding the detection of cyanobacteria, toxicity evaluation and enumeration. For example, these methods allow, together with gene sequencing, the identification of cyanobacteria species from environmental samples, with the advantage of removing the time needed for laboratory growth of the cyanobacteria cultures. Other studies have relied on genetic data obtained through DNA sequencing of cyanobacteria and cyanotoxins genetic markers in isolated cyanobacterial species and have subjected them to phylogenetic analyses in order to assess their genetic diversity, population structure, and in some cases, the phylogeographic structure and biogeographic pattern of the isolates [30,31,32]. DNA sequencing has made a major contribution in terms of publicly available gene sequences from both cyanobacteria and cyanotoxins in worldwide databases, with more than 80 genomes from cyanobacterial species having been sequenced so far. Apart from these genomes, genetic databases are being increasingly filled with new partial and complete sequences of DNA, all due to the growing use of molecular methods in cyanobacteria and cyanotoxin research.

PCR analysis is the most frequent technique used in the *in vitro* amplification of a DNA sequence by target specific primers. Cyanobacteria detection can be achieved by amplifying several genetic markers that have been developed for that specific purpose. To date, several primers have been designed and published to amplify: (a) common cyanobacteria specific DNA regions and; (b) genera specific regions for *Microcystis* and *C. raciborski* [33,34].

Similarly, toxicological evaluation through molecular methodologies has been made possible for the cyanotoxins microcystins, nodularin, saxitoxin, cylindrospermopsin and anatoxin-a with the detection of the genes involved in the gene clusters encoding these biosynthetic compounds. All of these gene clusters have already been completely sequenced allowing the designing of primers for their detection by conventional PCR assays, such as the multiplex-PCR assay developed for microcystins [35], cylindrospermopsin [36] and the singleplex PCR assays for saxitoxin [27], nodularin [26], anatoxin-a [29], and more recently, the multiplex-PCR for both microcystin and cylindrospermopsin from both cultured and field samples [37]. Another outcome for these developed primers is their application in the qPCR technique allowing the quantification of both cyanobacteria and cyanotoxins directly from an environmental sample. This is a more sensitive method in comparison to conventional PCR with detection limits of 8.8 cell/mL for *Microcystis* spp. and 258 cell/mL for *C. raciborskii* from field samples [38,39]. It also allows for the evaluation of the proportion and abundance of a given species as well as the establishment of the proportion of toxic and non-toxic genotypes within a cyanobacterial bloom [40,41].

Other molecular methods have also been applied to cyanotoxin and cyanobacteria research based on PCR technology such as the RAPD, RFLP and DGGE. Altogether, these methods allow the profiling of cyanobacteria and cyanobacterial communities directly from the environment, where they have been commonly used (for further information on these methods please see the review from Saker *et al*. [42]).

Non-PCR based methods are based on DNA probes that have been applied to cyanobacteria and cyanotoxin detection, and more recently, quantification. Some studies regarding DNA microarrays have been applied to gene expression in cyanobacterial species [43] and in the genetic profiling of natural communities [44]. Studies with this type of technology in cyanotoxins are recent and have been only applied to validation in the detection of microcystin (*mcyE*) and nodularin (*ndaF*) cyanobacteria producers in both strains and environmental samples [45]. Finally, FISH studies have been useful in the identification of cyanobacteria in natural samples [46,47] and also of the cyanotoxin microcystin (*mcyA*) in the cyanobacterium *M. aeruginosa* [48].

## 3. Phylogeny Studies in Cyanobacteria and Cyanotoxins

### 3.1. Genetic Markers

In cyanobacteria and cyanotoxin phylogenetic inferences, several genetic markers have been used (Table 1), all with the distinct purposes of being able to determine the amount of genotypes and/or lineages of the gathered isolates in order to assess their genetic diversity in a given location, their population or phylogeographic structure (Table 1). Other purposes are to resolve taxonomic issues among genera or at the species level, as well as in the establishment of biogeographic patterns (Table 1). Similarly to other studies that have used these markers, they were applied either separately or in a concatenated system (multi-gene analysis) using up to seven genetic markers [49,50]. The choice of the best phylogenetic marker is somewhat controversial since in some studies, which used more than one genetic marker, the results are conflicting [51], while in others, there is a clear agreement between all the applied markers [52,53,54]. Other studies have recently used a multi-gene analysis rather than a single genetic marker [49,50]. In those, the accuracy and reliability of the phylogenetic inference is increased since more genetic information can be analyzed [55]. Nonetheless, this type of analysis has its disadvantages since it requires more PCR reactions and particularly, more DNA sequencing, which in turn, makes it more expensive than when using a single genetic marker. Examples of the application of genetic markers to cyanobacteria and cyanotoxin phylogeny and biogeography studies will be presented.

**Table 1:** List of the genetic markers used and their respective application in phylogeny and biogeography studies of cyanobacteria.

Genetic marker	Taxonomy	Phylogenetic	Phylogeography	Evolution	Biogeography
*PC-IGS*	√	√	√		√
*ftsZ*		√			
*glnA*		√			
*gltX*		√			
*gyrB*		√			
*pgi*		√			
*recA*		√			
*tpi*		√			
*16S-23S ITS1-L*		√	√		
*16S-23S ITS1-S*		√			
*rpoB*	√				
*rpoC1*	√	√	√	√	
*nifH*	√	√	√	√	
*nifD*	√				
*16 rRNA*	√	√	√	√	√
*16S-23S ITS*	√	√	√		√
*rbcLX*	√				
*hetR*	√				
*psbA*		√			
*rbcL*		√			
*rbcS*		√			

### 3.2. Taxonomic Studies

Earlier in this review, we discussed the classification of cyanobacteria and established that the taxonomy was traditionally founded only on morphological characteristics. More recently, phylogenetic studies in cyanobacteria have applied genetic markers that aid in unraveling the association of related taxa through phylogenetic inferences. One of these examples is a study by Ishida *et al*. [56] where the 16S rRNA was used to determine the phylogenetic position of eight genera of Oscillatoriales that had an obscure definition. The data from this work showed that the Oscillatoriales group is not monophyletic and is genotypically heterogeneous and, therefore, suggested that their current taxonomic position should be revised. Wilmotte *et al*. [57], by studying the taxonomy of the genus *Oscillatoria*, also found it to be genotypically heterogeneous. Previously, Giovannoni *et al*. [58] studied cyanobacteria taxonomy by using the 16S rRNA marker and discovered that subsections II, IV and V are phylogenetically coherent while subsections I and III appear intermixed in the phylogenetic tree. Applying another genetic marker (*nif*H), Zehr *et al*. [59] demonstrated that subsections I, II, IV and V formed coherent groups. Later, Valério *et al*. [60], using the 16S rRNA marker in a global phylogeny of the six cyanobacterial orders, showed that the Nostocales, Stigonematales and Pleurocapsales form monophyletic groups while the Chroococcales and Oscillatoriales are polyphyletic.

Based on the phylogenetic analysis of the 16S rRNA, Otsuka *et al*. [11] proposed the unification of five species of the genus *Microcystis* into a single species of *M. aeruginosa* under the Rules of the Bacteriological Code. The five morphospecies were *M. aeruginosa*, *M. ichthyoblabe*, *M. novacekii*, *M. viridis* and *M. wesenbergii* in which DNA–DNA hybridization and phylogenetic data both showed no clear distinction among strains with the first method having more than 70% homology. The data from this work proved that all five species belong to the same species of the cyanobacterium *M. aeruginosa*. Previously, Neilan *et al*. [33], in a study of the genus *Microcystis*, found that this species is well separated from the other unicellular genera such as *Synechocystis*, *Synechococcus* and the filamentous genus *Nostoc* based on the information given by the 16S rRNA alone. By studying other genera, Henson *et al*. [10] tried to determine if *Nostoc* and *Anabaena* are separate genera based on information provided by the molecular marker *nifD*, since previous studies showed that other markers proved unable to differentiate these. Their results show that the two genera formed separate clades and, therefore, should be maintained as separate. In a subsequent study, Henson *et al*. [12] tried to establish the mono or paraphyletic nature of subsections IV and V of the cyanobacteria Bacteriological Code. Applying the genetic marker *nifD*, which was established in a previous study [10] to be a good candidate marker in resolving the heterocystous cyanobacteria lineage, they attempted to establish whether or not these two subsections are divided. Phylogenetic analysis showed that the heterocystous is monophyletic. However, with the use of *nifD*, the subsections IV and V formed two clades appearing intermixed, suggesting that their phylogenetic relationships do not reflect its classification.

A subsequent study by Gugger *et al*. [53], aimed at explaining the taxonomic relationships between strains of *Anabaena* and *Aphanizomenon*, with the prior knowledge that both genera are closely related although morphologically distinguishable. By using three distinct genetic markers (16S rRNA, 16S-23S ITS and *rbcLX*), the answers they obtained were that both genera did not form distinct clusters and appeared intermixed in the phylogenetic trees with high similarity values obtained between studied strains. This study showed that the taxonomy of both genera may be incorrect although morphological data supported their separation. The same observation was also made by Valério *et al*. [60] when using only the 16S rRNA marker.

Aiming to evaluate whether true branching is a monophyletic characteristic in the Order Stigonematales, Gugger *et al*. [61] conducted a study using the 16S rRNA applied to 16 strains of this cyanobacterial Order, which revealed a polyphyletic nature of the branching type since the three types of true branching were intermixed in the phylogenetic tree. In another study, Rajaniemi *et al*. [62] evaluated four genera of cyanobacteria belonging to the Order Nostocales (*Nostoc*, *Anabaena*, *Aphanizomenon* and *Trichormus*) on the basis of their genetic relationships along with morphological criteria. Their results revealed that the taxonomic classification should be revised since the planktonic *Anabaena*/*Aphanizomenon* and benthic *Anabaena* were not monophyletic having appeared in the three studied genetic markers intermixed much like the *Trichormus* strains. In view of the retrieved data, morphological criteria established by both botanical and bacteriological codes were not in agreement with the obtained genetic relationships for these three genera [62]. In *Anabaenopsis*, two strains were studied—*A. abijatae* and *A. elenkinii*—since *Anabaenopsis* spp. can morphologically resemble planktonic *Anabaena* and *Cylindrospermopsis* species [52]. The strains were gathered from Kenya, Mexico and Uganda lakes and tested with two distinct genetic markers (16S rRNA and the PC-IGS). Both phylogenetically gave the same tree topology, but while the 16S rRNA gave a high similarity value (97.5%), the PC-IGS gave a lower similarity value (93.6%) [52]. This in terms of sequence divergence resulted in different answers meaning that while using the 16S rRNA of the two strains indicated that they were the same species, the other genetic marker indicated that they were separated species. Alternatively, by using a multi-gene analysis consisting of four genetic markers (16S rRNA, *het*R, *nif*H and *rpo*C1), Thomazeau *et al*. [63] studied strains from the Sub-Saharan region in order to determine their contribution to the phylogeny of cyanobacteria. They found in their study the monophyly of Synechococcales, Chroococcales, Oscillatoriales (except the *Phormidium*-like lineage) and the Nostocales including the *Phormidium*-like lineage and the polyphyly of Pseudanabaenales [63]. Taxonomic revisions and erection of new taxa has become available in three recent studies from Komárek [13], Komarék & Mareš [14] and Zapomělová *et al*. [64]. All refer to the taxonomic revision of freshwater planktonic heterocystous cyanobacteria and the nostocacean genera should be based on the phylogenetic analysis of the 16S rRNA gene sequences, providing evidence that molecular methods are an important tool in the phylogenetic assignments of related taxa. Another work by Zapomělová *et al*. [64] describes how molecular methods can influence the erection of new taxa through gene sequencing of the 16S rRNA and phylogenetic analysis of the related taxa. In this last study, it was confirmed that *Anabaena bergii* and *Aphanizomenon ovalisporum* were transferred to the new *Chrysosporum bergii* and *Chrysosporum ovalisporum*, respectively. Also, in this work, the reclassification of *Anabaena tenericaulis* as *Dolichospermum tenericaule* was proposed.

### 3.3. Phylogenetic Studies

Determining the genetic diversity and population structure are the main aims in this type of study. In general, the research is focused on the collection of isolates of a certain cyanobacterial species and phylogenetic group to evaluate the population structure within a given lake, region or country, as well as to establish the genetic diversity (number of genotypes or lineages) of the isolates within the studied area. These studies also rely on the application of the genetic information of the isolated strains and compare them to their morphological characteristics. One of the most studied cyanobacteria genera is *Microcystis*. A study by Bittencourt-Oliveira *et al*. [30] is particularly relevant, where the genetic diversity of *M. aeruginosa* strains from a Brazilian reservoir was assessed and compared with their morphological characteristics. The study resulted in the formation of eight distinct genotypes and five lineages, which demonstrated the high degree of heterogeneity that this species carries in Brazilian waters based on the information of the phycocyanin intergenic spacer. Moreover, the results indicated that there was no correlation between morphotypes and genotypes with one genotype having more than one morphotype [30]. These results are in concordance with a subsequent study by Neilan *et al*. [65], where the genetic diversity for this genus was first established as heterogeneous since it formed several genetic lineages in the phylogenetic tree of the RFLP profiles. Other studies on *Microcystis* used other genetic markers and were carried out in other locations. These include the USA [66], Kenya and Uganda [54], Russia [67], Tunisia [68,69] and Japan [70,71]. In all, a high level of genetic diversity was observed with a high number of genotypes having been found. The heterogeneity of this genus is so well established that Wilson *et al*. [66] determined that the genetic variation between and among lakes is high with 53 out of 67 isolates shown to be genetically distinct. Other work with *Microcystis* relied on the separation of toxic from non-toxic forms assuming that phylogeny can be used as a tool to allow this separation without reverting to the traditional techniques that determine the toxicity of the isolates or strains. Some studies were conducted in this area such as those of Otsuka *et al*. [72] and Tanabe *et al*. [49]. The differences are that they first used a single genetic marker (16S-23S rRNA ITS) and they second used seven housekeeping genes targeting specifically *M. aeruginosa* in a MLST approach. In both studies, a clear separation of toxic from non-toxic strains of *Microcystis* was obtained, suggesting that both assays could be used in the assessment of a given strain or isolate as toxic or non-toxic for microcystins. Studies such as these have also been conducted in other cyanobacterium species such as *Anabaena circinalis* where the 16S rRNA marker was able to separate neurotoxic strains from non-neurotoxic strains [73]. In contrast, for toxic and non-toxic *C. raciborskii* cylindrospermopsin producing strains, Stucken *et al*. [74] demonstrated in a phylogenetic study that there was no relation between the phylogeny of the two genetic markers used (16S rRNA and 16S-23S ITS) and the presence of toxicity.

Phylogenetic studies in other cyanobacterium genera have also been carried out. Their main objective was to establish a correlation between genotypic data and phenotypes. Genera from the Order Nostocales have been also well studied such as *Nostoc* [75] and *Aphanizomenon* [76]. In the study of Svenning *et al*. [75], they aimed to demonstrate whether the 16S rRNA was able to distinguish symbiotic strains of this genus from free-living strains, and revealed that both types appeared intermixed in the phylogenetic tree. Using the genus *Aphanizomenon*, Wu *et al*. [76] assessed the morphological data and compared them with the genotypic data in Chinese freshwaters in order to determine the genetic diversity within this genus. Their data revealed that morphologically three strains of *Aphanizomenon*: *Aph. flos-aquae*, *Aph. issatschenkoi* and *Aph. gracile* were identified. The genotypic data was based on the information of three genetic markers that were analyzed separately and in a multi-gene analysis comprehending the 16S rRNA, *rbc*LX and *cpcBA*-IGS. The tree topologies for each gene were different but when using a concatenated system, the three *Aphanizomenon* strains that were identified grouped separately, something that was not observed when using either of the markers individually. Also, in an earlier study, Barker *et al*. [77] found that the genetic population of *Nodularia* in the Baltic Sea is heterogeneous with more than one genotype having been found. In *C. raciborskii*, a low genetic variability was observed when comparing isolates from several locations in Tunisia that appeared in a unique cluster with the use of the *rpoC1* marker [69,78].

### 3.4. Evolutionary Studies

In the Tree of Life, cyanobacteria are represented in the Bacteria group. In a study where the evolutionary relationships among cyanobacteria were evaluated, Giovannoni *et al*. [58] revealed that the deepest branches in the cyanobacterial phylogenetic tree were from *Gloeobacter violaceus* and two strains of the genus *Pseudanabaena*. Also, the unicellular and nonheterocystous filamentous cyanobacteria of subsection III appeared intermixed throughout the 16S rRNA phylogenetic tree suggesting that they might have multiple evolutionary origins [58]. Their data also suggested that heterocystous cyanobacteria may have diverged later in the cyanobacteria lineage. Also, Zehr *et al*. [59], based on the similarities of both *nif*H and 16S rRNA, arrived at the same conclusion. They have also proposed that the *nif*H genes responsible for nitrogenase genes were not obtained through horizontal gene transfer but by vertical transmission from an ancient ancestor. In another study, Neilan *et al*. [33] proposed the late divergence of the *Microcystis* genus due to high sequence conservation observed in the 16S rRNA. Turner *et al*. [79], in an in-depth evolutionary study of the cyanobacteria lineage and the 16S rRNA marker, determined that the most ancient descent of cyanobacteria is in fact *Gloeobacter violaceus*. They also confirmed the work of Nelissen *et al*. [80] that within cyanobacterial divergence the early origin of plastids was only preceded by two cyanobacterial genera, *Gloeobacter* and *Pseudanabaena* [80]. Evolutionary studies regarding the origin of cyanobacteria genera or species are scarce. Moreira *et al*. (unpublished data) conducted a study on the cyanobacterium *M. aeruginosa* and established that this species may have originated from the African continent. Studies such as these are scarce, but are nonetheless imperative.

The only work regarding the evolution of cyanotoxins is Rantala *et al*. [81] which established the early evolution of the cyanotoxin microcystins in contrast to the non-toxic species, and that these appeared as a result of the loss of ability to produce the toxin. Also in their work, they found that the nodularin toxin derived from microcystins. Evolution of other cyanotoxins is yet to be elucidated.

### 3.5. Phylogeographic Studies

Phylogeography allows the establishment of phylogenetic relationships within and among species from distinct geographic locations with the purpose of determining the presence or absence of spatial patterns. Studies based on the phylogeography of cyanobacteria genera or species are scarce and are based on the phylogenetic comparison of strains from diverse geographic origins. Some genera and species have been studied, namely *Aphanizomenon*, *C. raciborskii* and *M. aeruginosa*. Barker *et al.* [77] used the PC-IGS genetic marker to compare the population genetics of the *Aphanizomenon* in the Baltic Sea and in two North American lakes. Their data revealed that only a single genotype was found in the Baltic Sea *Aphanizomenon* colonies and that these are closer to the North American colonies. They also found that the two North American lakes analyzed had a higher genetic variation than the one observed in the Baltic Sea.

*C. raciborskii* is a cyanobacterium species that has received particular attention in regards to its phylogeographic structure due to its well-known invasive nature [82]. The five studies published to date all share the view that this species has a well-established phylogeographic structure. Dyble *et al*. [51] was the first to compare *C. raciborskii* strains from distinct geographic locations (Europe, Australia, America) by using two distinct genetic markers (*nifH* and *cpcBA*-IGS). Both gave a different clustering with *nifH* marker forming three separate clusters: Europe, Australia and America while the *cpcBA*-IGS marker formed two distinct clusters: Europe/Australia and America. Altogether, they were able to only differentiate the American isolates. In a subsequent study, Neilan *et al*. [83] published the phylogeography of this cyanobacterium species based on information from the 16S rRNA and cyanobacteria HIP1 PCR profiles. Although strains from the same continents were analyzed, similarly to the work of Dyble *et al*. [51], they established that the 16S rRNA marker in contrast to the HIP1 PCR profiles does not allow a good differentiation of the strains, especially at the species level. Nevertheless, they found two major groups being defined by the HIP1 PCR profiles that distinctly separate the American strains from the European and Australian strains. Their data also showed that in the Europe/Australia group, there was a high degree of genetic divergence. Later, Gugger *et al*. [84] increased the number of sampled continents for this species to four (Europe, Africa, Australia and America). Their conclusions based on information from the 16S-23S ITS marker showed that three main clusters existed: Europe, America and Australia/Africa. Haande *et al*. [85] also published a work using strains from the same four continents but utilizing three genetic markers (16S-23S ITS-L, nif*H* and PC-IGS) in a multi-gene analysis. The conclusions were the presence of three distinct clusters, with the strains of Africa being closer to the Australian strains, the American strains being more divergent and the European strains being closer to the Australian/African group [85]. In a more recent study, Moreira *et al*. [31] analyzed strains from all the main continents with a concatenated system of four genetic markers (16S rRNA, 16S-23S ITS-L, 16S-23S ITS-S rRNA and *rpoC1*) and identified three main clusters at a global scale: Europe, Australia/Asia and America/Africa.

*M. aeruginosa* is another cyanobacterium species that has been studied at a phylogeographical level. Neilan *et al*. [33] were the first to establish that the genera have no phylogeographic structure. By using the 16S rRNA marker, they found a high degree of genetic similarity (>91%) among strains from distinct geographic locations. Afterwards, Bittencourt-Oliveira *et al*. [30] applied the genetic marker *cpcBA*-IGS in their isolated strains from Brazil and compared them to strains from other geographic locations, namely Europe, North America and Japan, and found that these all appear to not form any distinct clustering among them and between the Brazilian strains. In a similar study, using two distinct genetic markers (PC-IGS and 16S-23S ITS), Haande *et al*. [54] analyzed the strains isolated from Uganda and Kenya with others from distinct geographic locations and found also that there is no well-defined phylogeographic structure, with the clusters consisting of strains from all around the world. Recently, by applying *M. aeruginosa* from all the main continents and through sequencing of DGGE bands of the 16S-23S ITS marker, van Gremberghe *et al*. [86] showed that the species carries in fact no phylogeographic structure and concluded that, at a biogeographic level, the species is considered to be cosmopolitan.

## 4. Biogeography Studies in Cyanobacteria and Cyanotoxins

Biogeography is a multidisciplinary science that aims to study living things in space and time [87]. Although the science has long focused on the distribution of animals and plants, microorganisms have only recently been debated. This is because microorganisms are easily dispersed globally, raising the question of whether they, like many macro-organisms, have historical biogeographies. Prokaryotic biogeography has been previously defined as the science that documents the spatial distribution of prokaryotic taxa in the environment at local, regional and continental scales [88]. Phenomena such as speciation, dispersal and extinction are proposed to explain the generation of biogeographic patterns [88]. Of all kinds of dispersal, passive dispersal is the most common to occur in cyanobacteria [89]. Vectors of passive dispersal for cyanobacteria include water bodies, fish, water birds, winds, airplanes, humans and scientific activities [89]. Biogeography studies involving cyanobacteria are generally based on the discovery of new genera or species in new environments. One of the studies published to date documenting the presence of cyanobacteria in extreme environments is that of Bahl *et al*. [32] in hot and cold desert cyanobacteria. Others such as the work of Strunecký *et al*. [90] describe the presence of *Phormidium autumnale* from ancient times in the Antarctic area, and others like Mazard *et al*. [91] and Ahlgren *et al*. [92] are based on the biogeography of the marine *Synechococus*, in North and South Atlantic gyres and Pacific Ocean waters, respectively. Regarding cyanotoxin biogeography, a single study by Moreira *et al*. [93] established that for *M. aeruginosa* microcystin-producing strains, the Asian continent appeared separated from the other continental groups and that those strains are closely related to European and North American strains.

The methods for biogeographic study in cyanobacteria and cyanotoxins are based on the identification and subsequent phylogenetic analyses of the isolates or environmental samples using predetermined genetic markers to establish their genetic diversity and geographical interaction. Examples include the studies of Taton *et al*. [94] and Ionescu *et al*. [95]. Both report the presence of cyanobacteria in extreme environments such as hot springs in Jordan [95] and in the East Antarctic lakes [94]. Other works report the presence of cyanobacteria species or genera in hot and cold deserts [32], namely in the public hot springs of Saudi Arabia [96], and also the presence of the cyanotoxin microcystin-LR in the cold Antarctic environment [97] and microcystin-LR and -RR in Saudi Arabian hot springs [96]. Reports of the presence of cyanobacteria in Artic and alpine habitats have also been described [98,99] as well as in hypersaline environments [100]. Besides the information on the presence of cyanobacteria and cyanotoxins in extreme environments, other studies describe how geography can influence cyanobacteria distribution at local or global scales. This is the case for *Nodularia* strains that were separately analyzed at a local scale (Baltic Sea) and at a global scale [101,102]. In the first study, a population structure was determined for the *Nodularia* strains isolated from nine stations in the Baltic Sea, which revealed the presence of two distinct genotypes that were not spatially equally distributed. In the second study, a worldwide comparison of *Nodularia* strains was conducted. In the work of Bolch *et al*. [101], through PCR-RFLP, RAPD and PC-IGS DNA sequencing, clustering revealed that the Australian *Nodularia* strains were distinct from the other locations worldwide (Europe and North America) and that between these are distinct populations of *Nodularia*.

Studies highlighting the impact of geography on cyanobacteria distribution are scarce but relevant for prokaryotic biogeography. One of these is the work of Papke *et al*. [3] where *Synechococcus* was screened in hot spring cyanobacterial mats from North America, Japan, New Zealand and Italy. From the data collected, it was observed that geographical isolation is the main reason why cyanobacteria diverged in these types of ecosystems since the same genotypes were found within and between countries. The data obtained from this study suggests that the central dogma “*everything is everywhere but nature selects*” is wrong since it denotes that there are no barriers for physical dispersion, where in contrast, this study shows that microorganisms are influenced by dispersal mechanisms. Other studies gave new insights in cyanobacteria biogeography, namely the works of Taton *et al*. [94] and Ionescu *et al*. [95]. In the study of Taton *et al*. [94] regarding the biogeography in the East Antarctic lakes, several cyanobacteria species were found to be endemic to those lakes while others were found to exist in other parts of the world, revealing the species’ capability of dispersal. In a similar study, Ionescu *et al*. [95] studied the cyanobacteria diversity of an isolated Jordan hot spring and found organisms equivalent to others found in thermal environments around the world, as well as endemic species. Since this particular ecosystem is completely isolated, the only possible explanation for this biogeographical finding is based on two previous biogeography theories: on one hand, the central dogma “*everything is everywhere but the environment selects*” could be true or on the other hand, the presence of chemical and physical barriers prevents the gene flow, making the organisms diverge from their ancestors since local speciation was found to occur [95].

Other more restricted studies about cyanobacteria biogeography involve the study of the worldwide distribution of a given cyanobacteria species. Garcia-Pichel *et al*. [103] found that the species *Microcoleus chthonoplastes* is a cosmopolitan species due to the low genetic diversity of the 16S rRNA sequences of the worldwide isolates. More recently, van Gremberghe *et al*. [86], also in a similar study, demonstrated the lack of phylogeographic structure in *M. aeruginosa* on a worldwide scale revealing that the species may have in fact a truly cosmopolitan distribution. Another study by Moreira *et al*. [31] about the worldwide distribution of *C. raciborskii* indicated that the European strains were probably colonized by strains from the Asian or Australian continents indicating the capability of dispersal that this species carries. Several hypotheses of dispersal have been proposed for the invasive *C. raciborskii* [82]. Initially, Padisák [82] proposed, based on documented reports, that the species had a first radiation center in the African continent and then dispersed to Indonesia and Central America and a second radiation center in Australia and that dispersion from there had then likely occurred by two routes: (1) an oceanic route to North and South America through birds or by unintentional human introduction; and (2) a continental route to Central Asia reaching the European continent also through birds and by river courses. Gugger *et al*. [84] later proposed a second hypothesis on the proliferation of this species suggesting that its recent invasion was not a spread either from Africa or Australia, but instead from warm refuge areas on each continent. The expansion to more temperate environments was proposed as follows: during the multiple glaciations and climatic changes of the Pleistocene, the species survived and the recent temperature elevation allowed the colonization of more areas. Later Haande *et al*. [85] suggested, based on the discrepancies in phylogeny, morphology and toxicology data that the spread of this species would have been rather through radiation within continents than the recent exchange between continents.

The studies presented so far report the distribution of cyanobacteria genera or species at a spatial level, but as stated initially, biogeography is the science that documents the distribution of a given taxa on spatial and temporal scales. For the latter, there is to date only one study from Bahl *et al*. [32] where they analyzed the biogeography of *Chroococcidiopsis* both at the temporal and spatial level in hot and cold deserts on a worldwide scale. Data from this work showed that the population is homogeneous through time and also, that the exchange between hot and cold deserts is a rare event. By using a calibrated microfossil, this study allowed a temporal scale to be added and also revealed that the central dogma that *everything is everywhere* is not correct since the same phylotype does not exist in both types of deserts, despite having a common ancestor. Also, the study reports that dispersal is not contemporary but ancient, and that it does not occur in the present day.

In light of the current knowledge, searching for cyanobacteria and studying their biogeography on a worldwide scale is the next step not only at the spatial level but also at the temporal level since it becomes imperative to establish whether or not microorganisms have patterns across space and time much like eukaryotes. Cyanobacteria biogeography is relatively recent and its incursion should not only be in extreme environments or distant regions such as the Polar Regions. Although relevant, the notion remains whether or not these microorganisms have, at a species level, a geographical segregation or as proved earlier, a cosmopolitan nature.

## 5. Conclusions

A compilation of the numerous studies in the field of phylogeny and biogeography for the cyanobacteria group and their produced cyanotoxins was the main goal of this review. The presented studies allow us to conclude that cyanobacteria phylogeny research has been restricted essentially to phylogenetic studies where the establishment of the genetic diversity of the isolates is the primary objective. Followed by taxonomic studies, the question of morphological classification of the genera or species was introduced into discussion from which the main conclusion is that the current taxonomic position of some genera should be revised. Evolutionary studies are poorly developed in cyanobacteria since most studies are about the origin and positioning of cyanobacteria within the Bacteria group. Though relevant, they pose the question of when the several cyanobacteria lineages arose in living history. Phylogeographic studies provided some light regarding the spatial patterning of the cyanobacteria genera and species around the globe. Though ubiquitous, some species of cyanobacteria were shown to have a spatial distribution in some of the studies, while in others, they were proven to be cosmopolitan. In this sense, biogeography is still in its infancy in cyanobacteria and cyanotoxin research. Though associated with the phylogeographic structure, this research aims to explain how this structure has come about in both time and space. Still limited to extreme areas of the world, recent publications have proven that much remains to be known about the occurrence and spread of cyanobacteria species as well as their related toxins, and to firmly establish if prokaryotic organisms are easily dispersed or have historical biogeographic patterns much like eukaryotes.
